# Spray-Drying Impact the Physicochemical Properties and Formation of Maillard Reaction Products Contributing to Antioxidant Activity of Camelina Press Cake Extract

**DOI:** 10.3390/antiox12040919

**Published:** 2023-04-12

**Authors:** Emilia Drozłowska, Małgorzata Starowicz, Natalia Śmietana, Urszula Krupa-Kozak, Łukasz Łopusiewicz

**Affiliations:** 1 Center of Bioimmobilisation and Innovative Packaging Materials, Faculty of Food Sciences and Fisheries, West Pomeranian University of Technology, Klemensa Janickiego 35 Street, 71-270 Szczecin, Poland; 2 Institute of Animal Reproduction and Food Research of Polish Academy of Sciences, Tuwima 10 Street, 10-748 Olsztyn, Poland

**Keywords:** antioxidant-rich ingredient, spray-drying, *Camelina sativa*, powdered food, gluten-free ingredient

## Abstract

Spray-drying is one of the most popular techniques in the food industry for converting liquid material from a fluid state into a form of dried particles to produce encapsulated or instant products. Instant products are considered as convenient foods; moreover, the goal of encapsulation is to close the bioactive compounds in a shell, preventing them from being affected by environmental factors. The purpose of this study was to examine the influence of spray-drying conditions, in particular three inlet temperatures, on the physicochemical and antioxidant properties of powders obtained from Camelina Press Cake Extract (CPE). The CPE was spray-dried at 140 °C, 160 °C and 180 °C. The solubility, Carr and Hausner Indexes, tapped densities and water activity of the powders were analyzed. The structural changes were also detected using FTIR spectroscopy. Additionally, the characteristics of the initial and reconstituted samples and their rheological properties were evaluated. The antioxidant potential, total polyphenols and flavonoids content, free amino acids, and the Maillard reaction products contents in the spray-dried powders were also evaluated. The results indicate a cascade of changes between the initial and reconstituted samples, and important changes in the bioactive potential of samples. The inlet temperature significantly influenced the solubility, flowability and particle sizes of the powders, as well as Maillard products formation. The results of the rheological measurements illustrate the changes after the reconstitution of extracts. This study indicates the optimal parameters of CPE spray-drying, those that yield favorable physicochemical and functional values, which may open up a promising path for CPE valorization, indicating its potential and the possibilities of its use.

## 1. Introduction

The population of people who suffer from gluten intolerance is growing significantly every year [1]. According to available reports, the spectrum of gluten-related disorders is broad, and includes several health problems such as celiac disease, wheat allergy and non-celiac gluten sensitivity (NCGS) [2]. The worldwide population of people diagnosed with celiac disease is estimated at 1.4%, and NCGS is estimated at 0.5–13% in the general population [3]. Patients on a gluten-free diet are highly exposed to oxidative stress, which is one of the factors related to the development of gluten-related disorders. According to Rowicka et al., the molecular mechanism of celiac disease pathogenesis is related to disturbances in oxidative–antioxidative balance caused by inflammation and oxidative stress [4]. Therefore, the diet for these patients should be enriched in antioxidant compounds.

Individuals on a gluten-free diet look for novel high-quality food products that, besides being gluten-free, will exhibit additional health-related properties, including antioxidant potential. One of the methods of improving the quality of the diet could be enriching food products with natural bioactive ingredients of plant origin, e.g., polyphenols, proteins and vitamins. Dietary polyphenols, which are natural bioactive compounds, have shown a protective role in intestinal disorders such as non-celiac gluten sensitivity, one of the gluten-related disorders [5]. These stable antioxidant agents could be introduced into the human diet as additives to foods.

*Camelina sativa* (L.) seeds are rich in phenolic compounds and exhibit an antioxidant potential [6]. According to Terpinc et al., the richness of phenolics in camelina seed, cake and oil is very high, and includes approximately 17 different chemicals, such as: protocatechuic acid, catechin, *p*-hydroxybenzoic acid, sinapine, ellagic acid, rutin, etc. [7]. The camelina seed also includes a high content of proteins, which is estimated at 23–30% [8]. It is reported that camelina exhibits a lower percentage of essential amino acids in comparison with canola protein [9], but its composition is well-balanced [10]. The increasing use of the cold-pressing procedure to obtain the oil is responsible for generating huge amounts of press cakes, which are currently considered a by-product [11]. The annual production of press cakes and oil industry residues in 2018/2019 was estimated at 600.47 million metric tons [12]. Due to this fact, there is a strong demand for novel solutions to edible press cake valorization [13]. According to the zero-waste concept and the circular economy approach, by-products should be recomposited or used as materials for new products. These statements are in line with novel strategies in the food sector, the higher awareness of consumers and the growing interest in “green” solutions. To reduce food waste, by-products represent a source of valuable extracts that serve as food additives, including for gluten-free diets. Their high water content and composition, however, makes fresh plant extracts prone to spoilage by microorganisms; in addition, individual phytochemicals tend to have low stability and show vulnerability to oxidation, degradation and hydrolytic cleavage [11,14]. Hence, they should be adequately stabilized before being stored and incorporated into the end product. Stabilization ideally should protect the base material against degradation, modify certain properties to make it easier to handle, mask off-flavors or odors, provide barriers among sensitive bioactive materials, and impart the ability to control the release rate of the phytochemicals [14].

Spray-drying is a widely used technique for the encapsulation of valuable compounds and the process of converting a material from a liquid form into the form of dried particles by atomizing it and evaporating water through contact between the aerosol and hot air [15]. This process is widely applied in the production of instant and convenient foods, two of the most rapidly growing areas of food production. It is also one of the main approaches to prolonging the durability of beverages, proteins, oils and bioactive compounds. Multiple plant extracts have been dried using this technique, such as flaxseed oil cake extract, mucilages, plant milks, etc. [15,16,17,18,19]. However, the drying process involves a high potential for stress factors that affect the quality of the dried material. In addition, the effect of this drying could alter the functional properties of the reconstituted extracts and their bioactive properties. Contrary to conventional drying, spray-drying is carried out as a more accurate technique for sensitive materials, such as emulsions or bioactive extracts, due to the very short contact time with hot air and the short duration of the drying process [20]. An adequate encapsulation method is one of the key factors to prevent bioactive compounds, and to prepare for further use compounds obtained from the various parts of plants and introducing them into food products.

Camelina Press Cake (CPC) contains a large amount of polysaccharides in addition to proteins, phenolic compounds and residual oil, which can act as a carrier or encapsulating material in the spray-drying process. According to Ibrahim et al. [21], CPC contains sucrose, lignin, mucilage, pectin and starch. It is important due to the fact that conventionally spray-dried powders are usually supported by carrier material such as maltodextrin or starch. In addition to its advantages, the spray-drying process also has a major impact on protein and polysaccharide structure and product properties such as water activity, solubility, protein structure and bioactivity. The utilization of CPC to produce spray-dried powders that could act as promising food additives in a gluten-free diet has not been reported so far. Moreover, to date, no study has been conducted to determine the influence of temperature on the physicochemical properties and formation of Maillard reaction products contributing to the antioxidant activity of Camelina Press Cake Extract (CPE) when subjected to spray-drying. The present study aimed to analyze the effects of spray-drying conditions—in particular, three inlet temperatures—on the properties of CPE after reconstitution. The main goal is the determination of the best inlet temperature, and a description of the key physicochemical differences between powders and samples after reconstitution. The antioxidant properties of reconstituted extracts could play a major role in the development the new additives for gluten-free foods, and open a pathway to obtaining ready-to-use products based on the CPC.

## 2. Materials and Methods

### 2.1. Materials

The Camelina Press Cake (CPC) derived from the cold-press process was donated by Olejarnia Niwki (Niwki, Poland). As per the manufacturer’s information, the approximate composition of CMP was as follows: solid content 80.50%, ash content 4.50%, protein content 41.97%, fat content 6.11%, carbohydrates 27.99%, and fiber 6.29%.

The following reagents were purchased from Sigma Aldrich (Sigma Aldrich, Darmstadt, Germany): sodium hydroxide, hydrogen peroxide, disodium phosphate, monosodium phosphate, 2,2-diphenyl-1-picrylhydrazyl (DPPH), methanol, Folin–Ciocalteu’s reagent, sodium carbonate, sodium chloride, gallic acid, acetic acid, sodium acetate, potassium ferricyanide, trichloroacetic acid, oxalic acid, 2,6-dichlorophenolindophenol, ferric chloride, ninhydrin, glacial acetic acid, cadmium chloride, glycine, ferric chloride hexahydrate and 2,4,6-tripyridyl-s-triazine (TPTZ), 2,2′-azino- bis(3-ethylbenzothiazoline-6-sulfonic acid) (ABTS), Ellman’s reagent (5,5′-dithiobis-2-nitrobenzoic acid), β-mercaptoethanol, trichloroacetic acid, urea, glycine, ethylenediaminetetraacetic acid (EDTA), tris(hydroxymethyl)aminomethane (Tris), sodium nitrite, aluminum chloride, lysine (Nα-acetyl-L-lysine) and quercetin. Glucose, hydrochloric acid, iron chloride and potassium hexacyanoferrate were delivered by Chempur (Chempur, Piekary Śląskie, Poland). The *o*-phtaldialdehyde used for fluorescence (OPA) and sodium dodecylsulfonate (SDS) were supplied by Fluka (Buchs, Switzerland). All reagents used in the study were of analytical purity and grades.

### 2.2. Obtaining the Camelina Press Cake Extract

**Figure 1:**
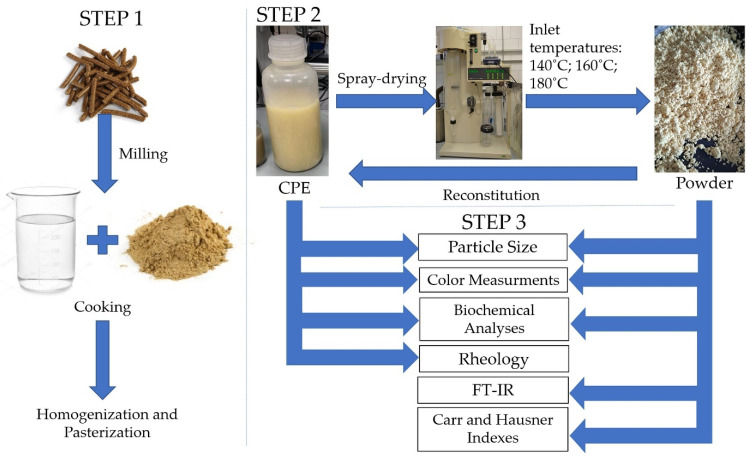
Scheme of research steps.

The CPC was milled (~150 mesh—approximately 0.10 µm) and mixed with distilled water to achieve a concentration of 2% *w*/*v*. The solution was heated for 60 min at a temperature of 80 °C with continuous stirring, and then the extract was homogenized. Finally, the CPE was pasteurized for 30 min at 60 °C and stored for further analysis (Figure 1).

### 2.3. Spray-Drying Protocol

The powders were produced by spray-drying using a lab-scale spray-dryer (Büchi B-290, Büchi Labortechnik AGT, Flawill, Switzerland). The following spray-drying air inlet temperatures were chosen: 140, 160 and 180 °C. The air outlet temperature was set at 55–60 °C. The obtained powders were stored in the darkness at 4 °C [22].

### 2.4. Powder Characterization

#### 2.4.1. Solubility in Water and Total Solid Content

For each sample, the total solid content was examined according to the AOAC (Association of Official Agricultural Chemists) standard method (no. 968.11) [23]. The solubility of the spray-dried sample was measured based on the previously described protocol [24] and the solubility of the powders was calculated according to the equation [25]:

$$Solublity=\frac{W_{2}-W_{0}}{W_{1}}\times 100$$

#### 2.4.2. Flowability and Cohesiveness of Powders

In order to estimate flowability and cohesiveness, firstly the bulk and tapped densities were calculated according to the methodology described [26]. In total, 5 g of each spray-dried powder was loaded into 25 mL tapped graduated cylinders. The tapped density was realized using the same protocol. The flowability and cohesiveness of all spray-dried powders were determined via the methodologies proposed by Carr [27] and Hausner [28], respectively. The Carr and Hausner indexes were calculated based on the following formulae:

$$CI(\%)=\frac{\rho_{t}-\rho_{b}}{\rho_{t}}\times 100$$

$$HR=\frac{\rho_{t}}{\rho_{b}}$$

#### 2.4.3. Water Activity Evaluation

The water activity (a_w_) values of powders were measured at 25 °C using MS1 Set-aw (Novasina, Lachen, Switzerland) equipment. Approximately 1 g of each powder was placed in the device, and stabilized for 30 min. Then, the measurements of a_w_ were conducted in triplicate.

#### 2.4.4. Determination of Powders Morphology

The surface morphology of the powders was observed using a scanning electron microscope (Vega 3 LMU, Tescan, Brno, Czech Republic) on the basis of a previously detailed protocol. The powder samples were placed directly on aluminum stubs with double-sided carbon conductive tape and coated with a thin gold layer using gold deposition. An accelerating potential of 15 kV was used during the microscopic observation [16].

#### 2.4.5. Reconstitution of Camelina Press Cake Extract (CPE)

To obtain the reconstituted CPE, the individual powders were mixed with distilled water to obtain a starting concentration, taking into account the Total Solids Content of each sample of powder. Each solution was mixed for 15 min (200 rpm). All measurements were carried out for the initial and reconstituted extract. The reconstituted samples are referred to as follows: AR (reconstituted CPE spray-dried at 140 °C), BR (reconstituted CPE spray-dried at 160 °C) and CR (reconstituted CPE spray-dried at 180 °C).

#### 2.4.6. Particle Size Distribution of Powders and Extracts

The particle size distributions of CPE before and after reconstitution were assessed using a Mastersizer 2000 (Malvern Instrument Ltd., Worcestershire, UK). The extracts dispersed in distilled water were stirred at a speed of 2000 rpm until an obscuration rate of 10% was obtained. The optical properties were defined as follows: refractive index 1.500 and absorption 1.00. The powder’s particle size distribution was determined using a dry sampling system—Scirocco 2000. Procedure (SOP): refractive index—1.52, vibration feed rate—50%, measurement time—10 s, dispersive air pressure—4 bar. The droplet size measurements Cn Be denoted as volume-weighted mean diameter D_4,3_ = _3i_d_4i_/i3_i_d_i3_ and D_3,2_ = 2d_i_d_3i_/i2_i_d_i2_ where n_i_ is the number of droplets with a diameter d_i_.

#### 2.4.7. Determination of pH, Titratable Acidity (TA)

Each powder was reconstituted and compared with the non-dried CPE. For each reconstituted sample, the pH and Total Acidity were measured. The pH was measured at 25 °C using a pH meter (CP-411, Elmetron, Zabrze, Poland). The TA of reconstituted beverages was determined by mixing the sample at 1:1 with the distilled water and titrating the solution with 0.01 M NaOH. The phenolphthalein (0.1%, *w*/*v* in 95% ethanol) was used as an indicator [29].

#### 2.4.8. Instrumental Color Determination

The changes of color in the CIE Lab color space both for powders and reconstituted samples were estimated. The samples were measured using a Konica Minolta CR-5 colorimeter with the Hunter LAB color system (Konica Minolta, Osaka, Japan). The main color coordinates are expressed as lightness (L*), redness/greenness (a*), and yellowness/blueness (b*). The Whiteness Index (WI), Yellowness Index (YI), Browning Index (BI) and total color difference (ΔE) were calculated using the following formulae [30]:

$$WI=100-{\left[\left(100-L^{*}\right)+a^{2}+b^{2}\right]}^{0.5}\;YI=142.86\times b\times L^{-1}\;BI=\left[100\times \left(\frac{a^{*}+1.75\times L^{*}}{5.645\times L^{*}+a^{*}-0.3012\times b^{*}}-0.31\right)\right]/0.17\;\Delta E={\left[{\left(L_{standard}-L_{sample}\right)}^{2}+{\left(a_{standard}-a_{sample}\right)}^{2}+{\left(b_{standard}-b_{sample}\right)}^{2}\right]}^{0.5}$$

### 2.5. Determination of Sulfhydryl Groups (–SH) and Disulfide Bonds (–S–S–) Contents in Spray-Dried Powders

The sulfhydryl groups (–SH) and the disulfide bonds (–S–S –) contents were determined following the methodology of Gong et al. [25]. In total, 180 mg of each powder sample was mixed with 30 mL of Tris-glycine buffer (0.086 M Tris, 0.09 M glycine, 4 nM EDTA, and pH 8.0) with 8 M urea. The solution was mixed for 30 min on a magnetic stirrer (150 rpm) and was then centrifuged at 6000 rpm for 10 min. Finally, the supernatants were collected for further analysis. In the aim of measuring the –SH content, 4 mL of supernatant was mixed with 160 µL of Ellman’s reagent (4 mg/mL). The absorbance of the mixtures was measured at 412 nm.

The next step aimed to determine the –S–S– content, and 8 µL of β-mercaptoethanol was added to 4 mL of the supernatant. The whole solution was incubated at 25 °C for 2 h and after this time, 10 mL of 12% trichloroacetic acid (TCA) was added. Next, the mixtures were again kept at 25 °C for 1 h and centrifuged at 6000 rpm for 10 min. The obtained precipitates were washed three times with 5 mL of TCA and dissolved in 6 mL of Tris-Glycine buffer. The precipitates dissolved with buffer were mixed with Ellman’s reagent and the absorbance of the samples was measured at 412 nm. The contents of –SH and –S–S– were calculated according to the formulae:

$$-SH(µmol/g)=\frac{73.53\times A_{412}}{C}$$

$$-S-S-(µmol/g)=\frac{Q_{1}-Q_{2}}{2}$$

### 2.6. Evaluation of Early, Advanced and Final Stage of Maillard Reaction (MR)

The content of available lysine, as an indicator of the early stage of MR, was determined as described by Michalska et al. [31] with modifications. Exactly 50 μL of the sample, 100 μL of OPA reagent, and 100 μL of water were added together and incubated for 3 min (96-well microplate; Porvair Sciences, Norfolk, UK). Then, the fluorescence reading was measured at λ_extinction_ = 340 nm and λ_emission_ = 455 nm using a microplate reader (Infinite^®^ M1000 PRO, Tecan, Switzerland). The quantitative analysis was performed via the external standard method, employing a calibration curve of Nα-acetyl-L-lysine.

The free intermediate compounds (FIC) were determined after sample extraction with 6% SDS and then recorded on a microplate reader (Infinite^®^ M1000 PRO, Tecan, Switzerland) set at fluorescence λ_extinction_ = 347 nm and λ_emission_ = 415 nm. Tryptophan fluorescence was measured at λ_extinction_ = 290 nm and λ_emission_ = 340 nm. The results are expressed in fluorescence intensity (FI) per mg of sample. FAST index data have been expressed as a percentage (%), as recently reported by Zieliński et al. [32].

The formation of brown pigments (melanoidins) was estimated as reported in detail by Zieliński et al. [32]. The measurements were performed in triplicate at 360 and 420 nm with the usage of a microplate reader (Infinite^®^ M1000 PRO, Tecan, Switzerland), and then were expressed as arbitrary absorbance units.

### 2.7. FTIR Analyses

The FTIR spectra of the powders were attained at room temperature via attenuated total reflection with an FTIR spectrometer (Perkin Elmer Spectrophotometer 100, Waltham, MA, USA). Each sample (100 mg) was scanned in a range between 650 cm^−1^ and 4000 cm^−1^ (40 scans and 4 cm^−1^ resolution). The obtained spectra were normalized, baseline-corrected and analyzed using SPECTRUM software (v10, Perkin Elmer Spectrophotometer, Waltham, MA, USA).

### 2.8. Rheological Measurments

The viscosities of the initial and reconstituted samples were evaluated using a rheometer (AR G2, TA Instruments Ltd., New Castle, DE, USA) with a stainless-steel cone plate geometry of 62 mm diameter and a 1° cone angle. The rheological properties were evaluated according to two protocols. The steady-state flow procedure was carried out in the range of 0.1 to 100 s^−1^ at a constant temperature (20 °C) and the temperature roadmap was established from 20 °C to 100 °C. The data of the rheological measurements were recorded using the TA Rheology Advantage Data Analysis equipment software V 5.4.7.

### 2.9. Determination of the Reducing Sugars Content (RSC), Total Free Amino Acids (TFAA), Total Polyphenols Content (TPC) and Total Flavonoids Content (TFC)

The Reducing Sugars Content of samples was determined by the DNS (3,5-dinitrosalicylic acid) protocol as previously described [33]; 1 mL of each reconstituted powder and the initial sample was mixed with 1 mL of 0.05 M acetate buffer (pH 4.8) and 3 mL of DNS reagent and vortexed. The samples were boiled for 5 min and then cooled down to room temperature. The absorbance value was measured at 540 nm using a microplate reader (Synergy LX, BioTek, Winooski, VT, USA) in the 96-well microplate. Glucose in acetate buffer was used as a standard to prepare the calibration curve.

Total free amino acid levels (TFAAL) were analyzed based on the ninhydrin method with a Cd-ninhydrin reagent as previously described [34]. For the measurements, 1 mL of each sample was mixed with 2 mL of a Cd-ninhydrin reagent. The samples were shaken, then heated at 84 °C for 5 min and finally cooled down. The absorbance was determined at 507 nm. Finally, the results were expressed as mg Gly per gram of sample in accordance with the standard curve based on the glycine, and with respect to the dilution factor.

The Total Polyphenol Content (TPC) and the Total Flavonoids Content (TFC) were determined as described by Tong et al. [35]. The TPC analysis was conducted following the Folin–Ciocalteu method. Firstly, 100 µL of the sample, 6 mL of distilled water and 0.5 mL of Folin–Ciocalteu reagent were mixed. After 3 min of incubation, 1.5 mL of saturated Na_2_CO_3_ solution was added. Finally, the mixtures were incubated for 30 min in the dark at 40 °C. The absorbance was detected at 765 nm. The Polyphenols Content of samples was expressed as mg gallic acid equivalents (GAE) per mL of sample (mg GAE/mL).

Aiming to measure the TFC, 250 µL of supernatant was mixed with 1 mL of distilled water and 75 µL of 5% NaNO_2_ solution. After 5 min incubation, 75 µL of 10% AlCl_3_ solution was added to the solution, and next the mixture was left to incubate for 6 min. After this step, 250 µL of 1 M NaOH was added. The total volume of the sample was filled up to 3 mL with distilled water. Then the absorbance was measured at 510 nm. Quercetin was used for a calibration curve, and the results have been expressed as mg of quercetin equivalents (QE) per mL of the sample (mg QE/mL) [24].

### 2.10. Changes in Antioxidant Proprities

To determine the antioxidant properties of the CPE, as well as the DPPH and ABTS radicals scavenging activities, Reducing Power (RP) and FRAP tests were chosen. The DPPH radical scavenging activity was determined by mixing 1 mL of each reconstituted powder solution with 1 mL of 0.01 mM DPPH methanolic solution. Then, the absorbance was determined at 517 nm. The ABTS was determined according to the previously adjusted method [16]. For ABTS scavenging activity determination, 50 µL of each sample was mixed with 3 mL of diluted ABTS^+^. The absorbance was measured at 734 nm after 6 min of initial mixing.

Reducing Power (RP) was measured by mixing 500 µL of the sample with 1.25 mL of phosphate buffer (0.2 M, pH 6.6) and 1.25 mL of 1% potassium hexacyanoferrate. Then, the solutions were incubated at 50 °C for 20 min before the addition of 1.25 mL of trichloroacetic acid. The samples were centrifuged at 3000× *g* for 10 min (Centrifuge 5418 Eppendorf, Warsaw, Poland) and yielded 1.25 mL of the supernatant. Then, 0.25 mL of 0.1% iron chloride was added, and finally the absorbance was measured at 700 nm to determine the reducing power [22].

For the FRAP measurements, 25 mL of acetate buffer (300 mM), 2.5 mL of 2,4,6-tripyridyl-s-triazine (TPTZ) solution (10 mM in 40 mM HCl) and 2.5 mL of ferric chloride hexahydrate aqueous solution (20 mM) were mixed. To 300 μL of FRAP reagent in a microcentrifuge tube, 10 μL of extracts were added and vortexed for 10 s. The absorbance was measured at 593 nm [36].

ABTS, DPPH and RP were calculated according to the standard curve based on the Trolox reagent. FRAP was expressed as ascorbic acid equivalents (AAE) per mL of a reconstituted sample using the standard curve for ascorbic acid.

### 2.11. Statistical Analyses

The results of the present study have been expressed as mean ± standard deviation and were subjected to a one-way analysis of variance (ANOVA) test with the software Statistica 13.0 (StatSoft, Kraków, Poland). Significant differences between means were determined using Fisher’s LSD (Least Significant Difference) NIR multiple comparison tests at *p* < 0.05. All experiments were conducted in three replications.

## 3. Results and Discussion

### 3.1. Powders Characteristics

Table 1 presents the results concerning the Particle Size Distribution, Total Solid Content (TSC), Hausner Ratio (HR) and Carr Index (CI) of the powders. These results demonstrate the flowability and cohesiveness of the powders. The aim of evaluating the Carr Index is to indicate the compressibility of a powder. The obtained results have been compared with the scale proposed in the previous study, and based on that, favorable parameters were exhibited by sample A—140 °C (HR—1.28 ± 0.04 and CI—26.83 ± 0.61%), and disadvantageous parameters by sample C, which was spray-dried with an inlet temperature of 180 °C (HR—1.58 ± 0.12 and CI—37.63 ± 6.08%) [24]. It was observed that the powders exhibited particle size tendency that was reduced in connection with the decrease in flowability of the obtained powders. A similar tendency was observed by Reddy et al. [37] during the spray-drying process of goat’s milk. The authors observed that the weak and unstable properties of dairy powders associated with small particle sizes may be related to the large surface area relative to the weight of the powder. It was highlighted that a larger contact area between powder particles results in greater accessibility for cohesive forces that can resist flow. The authors also mentioned that this phenomenon was previously described by Fitzpatrick et al. [38].

**Figure 2:**
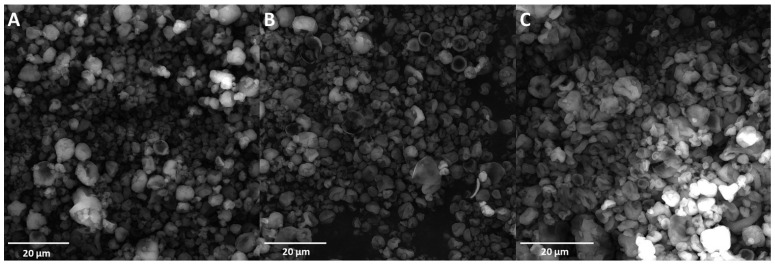
SEM images of CPC spray-dried powders. (**A**)—sample spray-dried at 140 °C; (**B**)—sample spray-dried at 160 °C; (**C**)—sample spray-dried at 180 °C.

The tapped bulk density decreased with the increase in spray-drying inlet temperature, and minimized the particle size values. This was affected by a higher concentration of powders and a lower content of occluded air in the powder particles, as well as a higher final density of the samples. A similar tendency was reported by Reddy et al. [37], as well as by León-Martínez et al. [15]. Figure 2 shows the SEM images of spray-dried CPE particles.

Particles dried at the three temperatures exhibited a relatively spherical shape, with cavities typical of spray-dried materials resulting from particle shrinkage due to the rapid evaporation of water [17]. In addition, the SEM micrographs indicated that the outer surfaces of the dry particles were free of cracks and disruptions. A similar morphology of powders was observed by Bustamante et al. when mucilages (from chia and flaxseed), as well as inulin, were used as encapsulating agents [17]. In our previous studies, a more wrinkled and folded morphology was observed when Flaxseed Oil Cake Extract was subjected to spray-drying [16,39]. In contrast, a spherical shape with holes and a very collapsed morphology was observed in spray-dried whey protein isolate powders, which can be linked with the surface activity of whey protein [40]. The most uneven morphology could be observed in sample C spray-dried at 180 °C. The particles also exhibited a tendency towards aggregation, and this observation is consistent with the results regarding the Carr Index and Hausner Ratio determination. A tendency towards agglomeration was also observed by León-Martínez et al. in spray-dried nopal (*Opuntia ficus-indica*) mucilage, which can be attributed to the static electrical effects and van der Waals forces that result in agglomerates consisting of individual grains of material linked by submicron dust (of the same material), while the agglomerated structures can also bind to each other [15].

**Figure 3:**
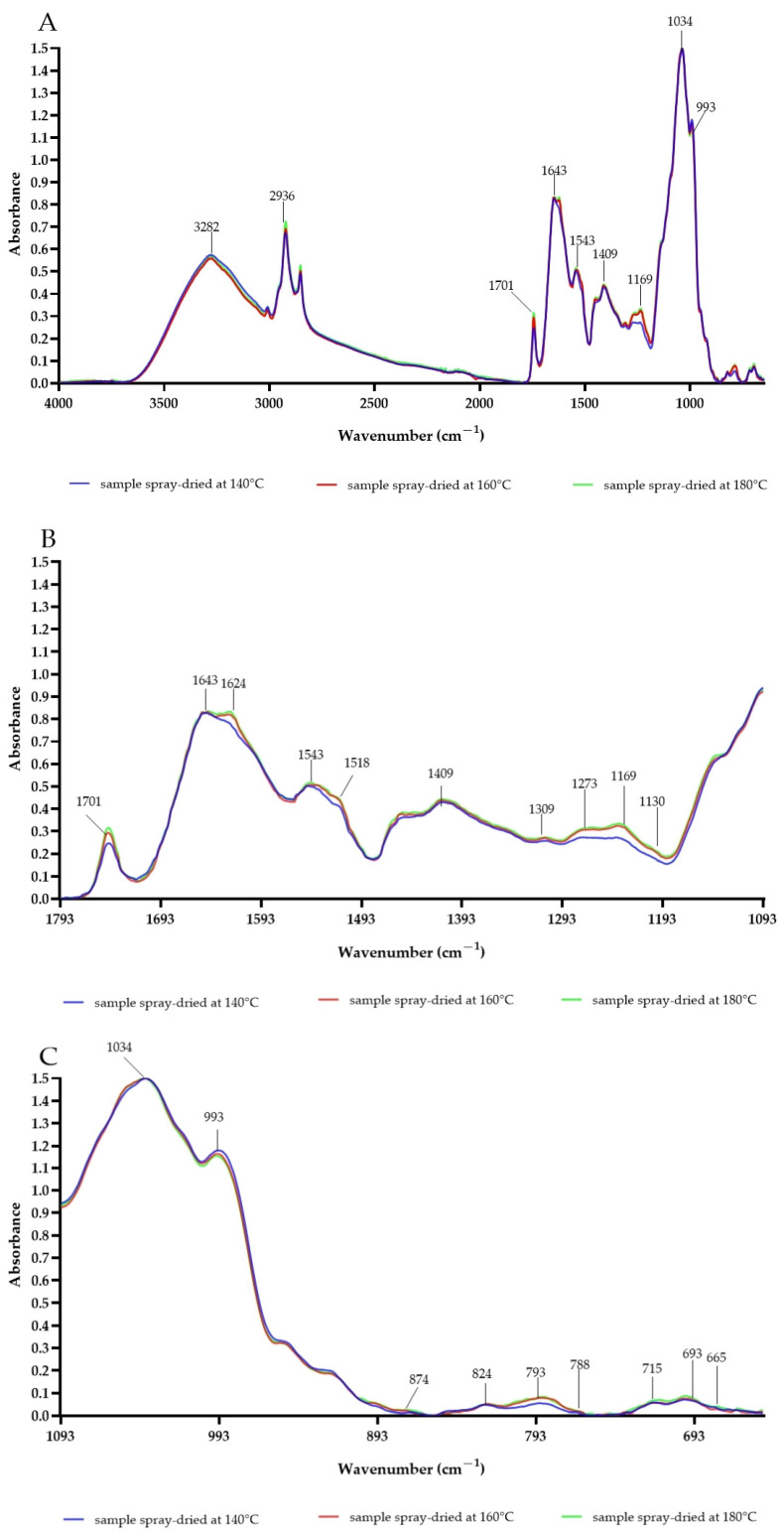
The FTIR spectra of experimental powders obtained at different spray-drying conditions. (**A**): Whole FTIR spectrum; (**B**): differences between samples in area from 1793 cm^−1^ to 1093 cm^−1^; (**C**): differences between samples from 1093 cm^−1^ to 650 cm^−1^.

FTIR spectroscopy was exploited to compare the chemical compositions and secondary structures of the CPE after spray-drying, as shown in Figure 3. Each powder presented a characteristic bond at 3282 cm^−1^, which could be associated with N–H stretching vibrations of the primary amide structure, as well as O–H stretches, C–H stretches, and residual water, similar to those seen in FOCE (Flaxseed Oil Cake Extract) powders [16,25,41]. Spray-drying shifted the amide I and amide II bands to lower energy levels in samples B and C. The amide I peak shifted from 1643 cm^−1^ (sample A) to 1624 cm^−1^ (samples B and C). Similarly, the amide II peak shifted from 1543 cm^−1^ (sample A) to 1518 cm^−1^ (samples B and C). These bands may be connected with C=O stretching vibrations, N–H stretching vibrations, N–H bending vibrations, and C–N bending vibrations in proteins [16,41]. The observed changes can be attributed to the shrinkage of the protein secondary structure, including its unfolding with a broader range of conformational geometry upon spray-drying [42]. According to Boostani et al. [43], changes in areas could also occur in relation to the shift base products of the Maillard reaction. Similar amide I and amide II peak position shifts have been reported by Fu et al. for soy protein isolate modified by the Maillard reaction [44]. The results in other studies also indicate that peaks in the area from 1700 cm^−1^ to 1600 cm^−1^ and differences between them could be associated to C=O stretching bands of Amadori compound products or the Schiff base imine group. The peak at 1701 cm^−1^ was the highest for sample C [43]. The peak at 2936 cm^−1^ responsible for characteristic CH_3_ and CH_2_ was higher for samples spray-dried at 180 °C. According to Li et al., the strong and broad vibrations between 2500 and 3600 cm^−1^ may be related to the glycosidic ring of Camelina mucilage [9]. Peaks at 1034, 993 and 820 cm^−1^ also could be due to mucilage. The bound at 1034 cm^−1^ could be a signal from the galactose in the mucilage included in the CPE [8]. It was observed that the sugars were stable during the spray-drying process. There were also differences between the samples in the range of 1293–1193 cm^−1^. The absorbance increased in accordance with increases in the inlet temperature. This area in connected with amide bond III, and reveals C–N stretching and N–H bending vibrations [43]. The increase in this area could be coupled with the effects of the Maillard reaction and the rebuilding of protein structures. Moreover, an increase in peak 793 cm^−1^ with increasing temperature suggests R_1_R_2_C=CH_2_ group production via the Maillard reaction, which was also reported for modified soy protein isolates [44].

The results of the evaluation of solubility and water activity (a_w_) are presented in Table 2. The increase in inlet temperature strongly affected the solubility of the obtained powders. In the case of sample B, it could be observed that an inlet temperature of 160 °C allowed for the highest solubility (70.74 ± 0.81%). This parameter decreased significantly when the inlet temperature rose up to 180 °C. A negative effect of increasing the inlet temperature on the solubility of the obtained powders was also observed by Fang et al. in the case of the spray-drying of milk concentrates [45]. Similar tendencies were previously observed for Flaxseed Oil Cake Extract (FOCE) [16]. The solubility could be connected to the partial denaturation of the proteins included in spray-dried CPE [16]. The results of water activity (a_w_) are presented in Table 2. It is well-known that, to avoid microbiological contamination, an a_w_ value of less than 0.7 is recommended. In the present study, the a_w_ value was even lower than recommended (Table 2). In comparison with the spray-dried FOCE [36], the results of a_w_ determined for CPE were lower, but were similar to those obtained by Liu et al. for spray-dried skim milk (a_w_ = 0.25) [46].

The changes in sulfhydryl group (–SH) and disulfide bond (–S–S–) contents are also presented in Table 2. A significant increase in the contents of the –SH– and –S–S– groups between spray-dried variants was observed. The content of disulfide bounds for sample A was lower than that in the ones spray-dried at 160 °C (sample B) and at 180 °C (sample C). The results regarding the –SH were statistically significant for all samples (*p* < 0.05). The results of –S–S– content were also statistically different for each sample (*p* < 0.05). Similar results were described by Gong et al., who observed the highest content of –S–S– after a spray-drying process [25]. The process of breaking the disulfide bond could be induced by heating, thus contributing to the increase in sulfhydryl groups. Sulfhydryl groups are part of the tertiary structure of proteins and are also involved in weak secondary bonds. Changes in –SH content are indicative of protein denaturation while drying. The recorded changes suggest that spray-drying caused changes in the protein structure, which could be due to the partial denaturation of proteins included in the CPE. These changes affected several parameters, such as the functional characteristics of the proteins or the solubility of the powders [45].

The results of the evaluation of the early-, advanced- and final-stage Maillard reaction (MR) products are presented in Table 3. Statistically significant (*p* < 0.05) changes in the availability of lysine, Fluorescent Intermediate Compounds (FIC), tryptophan (TRP) and melanoidins were observed, and were associated with the increase in the inlet temperature. According to Starowicz and Zieliński, this could be connected with nutritional losses due to the formation of new molecules from lysine-free amino residues and reducing sugars [47]. A slight difference between the FAST results affected by inlet temperature was observed, although they were not statistically important (Table 3). However, a positive observation was made in relation to experimental powders in comparison to the cookies with rutin that were previously studied, wherein the FAST values were much higher (ranging from 250% to 1400%) [48]. On the other hand, the formation of browning pigments (melanoidins) was observed. Melanoidins exhibit several beneficial health-related properties, such as chemoprevention and antimicrobial and antioxidant capacities [48]. At both wavelengths of 360 and 420 nm, the highest level of melanoidins was determined in powders spray-dried at 140 °C, followed by those dried at 160 °C and those at 180 °C. The use of mixed matrices containing proteins and polysaccharides increased the bioactivity of the reconstituted samples through the formation of Maillard reaction products [49]. The available reports indicate that Maillard conjugates exhibit an antioxidant activity in spray-dried materials [30,50,51].

### 3.2. Characterization of the Samples before and after Reconstitution

In order to evaluate the changes manifested by the application of various inlet temperatures, the extracts were reconstituted and compared with the initial samples. As presented in Table 4, the physicochemical properties changed after the spray-drying. The changes in the pH were statistically significant (*p* < 0.05) for every sample. A slight increase in the pH and the TA after reconstitution could be observed. Similarly, an increase in acidity was also observed for carob juice by Ali et al. [50]. The authors explained this phenomenon via the release of sugars during the spray-drying process, which could be confirmed by the statistically significant increase in the Reducing Sugars Content (RSC) (*p* < 0.05). The samples spray-dried at 160 °C and 180 °C had the highest RSC values, and these samples also exhibited a higher acidity.

In the case of the TPC and TFC, statistically significant differences were also observed (*p* < 0.05). The results regarding polyphenols contents were only slightly different between variants. The decrease in flavonoids between CPE and spray-dried samples was greater. This observation is in agreement with those of Wu et al., who observed the significant impact of spray-drying on polyphenols and flavonoids [51]. The increase in the polyphenol content in samples AR and BR in comparison to sample CR could have been caused by the rebuilding of several chemical structures [52]. An overall decrease in TPC was observed by Wilkowska et al., who stated that after the spray-drying, approximately 73% of polyphenols could be lost [20].

Statistically significant differences in the Total Free Amino Acids (TFAA) were observed. The initial content of TFAA in the CPE was estimated in 5.09 ± 0.04 mg Gly/mL. The spray-drying process caused a significant increase in the TFAA (*p* < 0.05). The highest content of TFAA was observed in sample CR (7.14 ± 0.01 mg Gly/mL). This could be connected with the previously described changes in protein structure and in sulfhydryl group (–SH) and disulfide bond (–S–S–) contents, which were caused by the partial denaturation and rebuilding of proteins. Thus, the obtained results are in accordance with the FTIR analyses results (Figure 3), wherein the highest differences were observed in the amide bounds, and the peaks for polysaccharides and sugars from mucilage were similar. Additionally, the obtained results regarding TFAAL are in agreement with those presented in Table 3, wherein a higher capacity for lysine and tryptophan was observed. This could have been affected by the Maillard reactions. Partial hydrolysis resulting in an increase in free amino acids was also reported in our previous studies, in which Flaxseed Oil Cake Extract was subjected to spray-drying [30]. In particular, in the system of spray-dried emulsion containing flaxseed oil, an increase in the contents of free amino acids that could enter into Maillard reactions contributed positively to the oxidative stability of the encapsulated oil [30]. An increase in the TFAA and Maillard reaction products was also noted by Rodrigues et al. when recovering antioxidant protein hydrolysates from shellfish waste streams via subcritical water extraction [53]. The authors also attributed this phenomenon to partial hydrolysis caused by a high temperature.

The results of changes in the bioactivity of the reconstituted samples in comparison with the initial samples are summarized in Table 5. The ABTS and DPPH scavenging activities were higher after the spray-drying in comparison to the non-dried sample. The polyphenols oxidation process observed during the spray-drying process induced a higher scavenging activity in the samples. According to Mrkìc et al. [54], partially oxidated polyphenols may increase the radical scavenging activity more notably than non-oxidized polyphenols. The FRAP and RP results indicate the significant impact of the inlet temperature on the capacity for ferric reduction. The lowest values were observed for sample CR (FRAP—3.94 ± 0.05 mg AAE/mL and RP—2.29 ± 0.01 μmol Trolox/mL). Similarly, a decrease in FRAP during spray-drying was also mentioned by Papoutsis et al. for dried lemon by-product aqueous extracts [55]. The authors suggested that these effects could be caused by the temperature-based degradation of bioactive compounds. The impact of the chosen inlet temperature on the antioxidant activity was previously observed for FOCE, when a significant decrease in DPPH and ABTS between the variants spray-dried at 160 °C and 180 °C was also observed [16].

The results of the color measurements are summarized in Table 6. It was noted that the highest L* value and WI index were obtained for sample A, which was spray-dried at 140 °C. In contrast, as the inlet temperature increased, the aforementioned values decreased. According to Chen et al., the increase in particle size was likely due to aggregation, and resulted in color changes, such as the appearance of darker, more yellow and red powder colors [56]. Similar results were obtained by Brishti et al., who identified less red and yellow hues in spray-dried powders [57]. The authors highlighted a connection between the decreasing particle size and the greater lightness of powders [57]. The results obtained were in partial agreement with those obtained for FOCE, for which it was noted that the spray-drying process leads to a reduction in L* [16]. Additionally, the changes of color between the samples could be an effect of the thermal breakdown of glucosides from CPE during the spray-drying. The changes in BI can indicate the effects of non-enzymatic browning in the powders [58]. Slight but statistically significant changes in the Browning Index (BI) were observed (*p* < 0.05). It was noted that the BI was highest in the case of sample C. Similar observations were described by Koca et al., who observed the effect of the inlet temperature on the final product, and also pointed out the effect of high temperatures during the spray-drying process on the formation of Maillard reaction products and the final values of the powders’ Browning Index [59].

**Figure 4:**
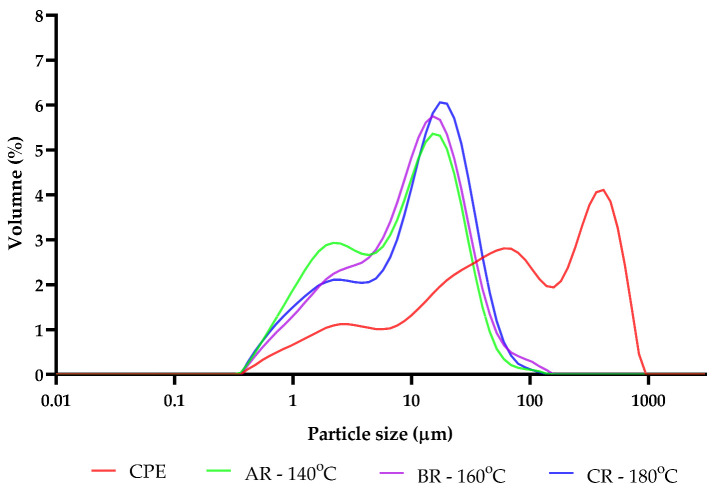
The distribution of the particle sizes of initial and reconstituted samples. CPE: Camelina Press Cake Extract; AR—140 °C: reconstituted sample spray-dried at 140 °C; BR—160 °C: reconstituted sample spray-dried at 160 °C; CR—180 °C: reconstituted sample spray-dried at 180 °C.

It could be observed that the lightness (L*) of reconstituted samples increased in accordance with the increase in the inlet temperature. Additionally, the ΔE also increased, and the highest content of this parameter was recorded for sample CR, which was spray-dried at the inlet temperature of 180 °C. The highest YI and an increase in b* were also observed in this sample. The observed changes may be related to the formation of Maillard reaction products [16]. The presented results could be related to the increases in RSC and TFAA contents, which were the results of polysaccharide and protein degradation during the spray-drying process. These results are in line with the particle size changes, because the particle sizes of the obtained powders decreased after the spray-drying, and the particle sizes of the reconstituted samples were also smaller than their initial values (Figure 4). As the inlet temperature increased, the particle sizes of the reconstituted samples increased slightly.

### 3.3. Rheological Changes before and after Reconstitution

**Figure 5:**
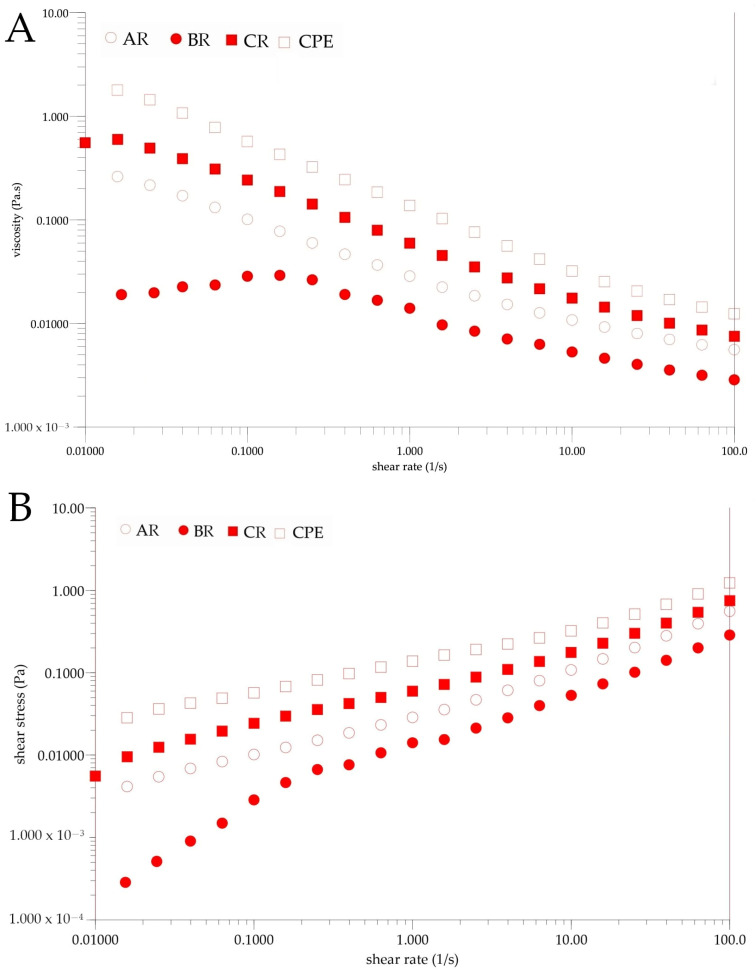
Rheograms of CPE and reconstituted samples. (**A**)—Changes of viscosity. (**B**)—Changes of shear stress. CPE: Camelina Press Cake Extract; AR: reconstituted sample spray-dried at 140 °C; BR: reconstituted sample spray-dried at 160 °C; CR: reconstituted sample spray-dried at 180 °C.

**Figure 6:**
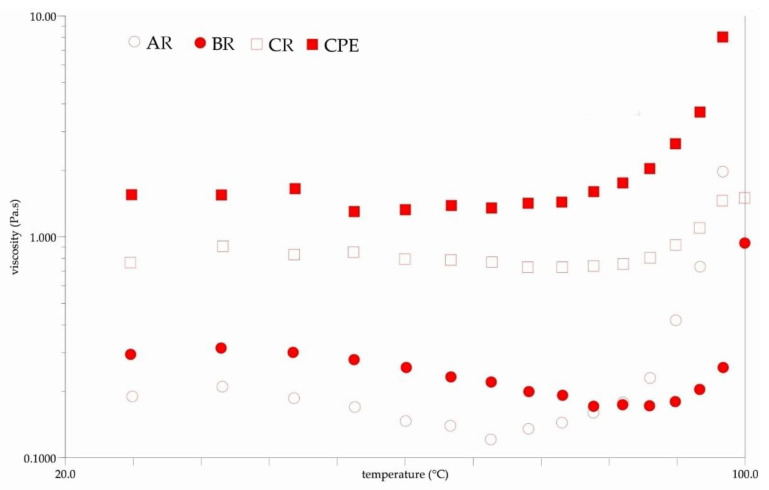
The viscosity changes during the increase in temperature. CPE: Camelina Press Cake Extract; AR: reconstituted sample spray-dried at 140 °C; BR: reconstituted sample spray-dried at 160 °C; CR: reconstituted sample spray-dried at 180 °C.

Figure 5 shows the results of the rheological tests. It can be seen that each sample exhibited a non-Newtonian behavior before and after reconstitution (Figure 5B). Viscosity could be influenced by several factors, in particular by the intermolecular strength between water-soluble molecules (sugars, acids and other macromolecules). These interactions result from intermolecular distances and bonding forces, which are strongly influenced by temperature and sample concentration [60]. Changes in the compounds’ contents in the reconstituted samples might have affected the obtained viscosity. The observed viscosity was generally lower in the reconstituted sample than in the initial sample. Differences caused by inlet temperatures were also observed. It was observed that the viscosity of the BR sample was lower than that of the AR and CR samples. The observed non-Newtonian behavior of the samples is typical for fluids containing natural mucilage. Similar behaviors were observed by León-Martínez et al. for reconstituted *Opuntia ficus-indica* mucilage solutions and in previous research on Flaxseed Oil Cake Extract (FOCE) [15,24]. The same phenomenon was observed when measuring viscosity changes at different temperatures. As can be seen from the temperature roadmaps (Figure 6), the viscosity decreased with the increasing temperature. A similar phenomenon has been observed by several authors, and these results could be explained as the effect of an increase in the thermal energy of the molecules, which increases their mobility and the inter-particle distances [15,61,62]. 

## 4. Conclusions

The spray-drying conditions influenced the physicochemical and functional properties, as well as the bioactivity, of the samples prepared in the presented study. The obtained powders exhibited a low water activity and were characterized by different solubility properties depending on the spray-drying conditions. It was also observed that the results of the Maillard reactions enhanced the antioxidant potential of the extract. The powders spray-dried at 140 °C exhibited the highest antioxidant potential, but weaker functional properties than powders obtained at 160 °C. The presented results indicate that the best inlet temperature for CPE spray-drying is 160 °C. The highest temperature caused the greatest loss of antioxidant compounds and a weak flowability and cohesiveness in the powders. Powdered extracts of CPC could be an attractive and valuable additive in gluten-free food due to their high antioxidant potential. The obtained results could be a pathway to developing novel, ready-to-use drinks and functional food additives. Moreover, they could show high potential in the nutraceutical and food additives areas, and the proposed solution also adheres to a circular economy approach.

## Figures and Tables

**Table 1:** The effect of spray-drying conditions on tapped bulk density, flowability, cohesiveness, particle size and Total Solids Content (TSC) of experimental powders.

Sample	Tapped Bulk Density (g/cm^3^)	HR	CI (%)	D_4;3_ (µm)	D_3;2_ (µm)	TSC (%)
A—140 °C	0.382 ± 0.022 ^a^	1.28 ± 0.04 ^a^	26.83 ± 0.61 ^ab^	54.36 ± 1.27 ^a^	10.95 ± 1.09 ^a^	93.53 ± 0.45 ^a^
B—160 °C	0.362 ± 0.040 ^a^	1.37 ± 0.07 ^a^	27.50 ± 0.54 ^ab^	51.81 ± 0.55 ^a^	12.17 ± 0.32 ^b^	95.70 ± 0.19 ^a^
C—180 °C	0.364 ± 0.027 ^a^	1.38 ± 0.12 ^b^	31.63 ± 1.08 ^b^	41.36 ± 4.30 ^b^	8.55 ± 0.09 ^c^	97.29 ± 0.29 ^a^

A—140 °C: sample spray-dried at 140 °C; B—160 °C: sample spray-dried at 160 °C; C—180 °C: sample spray-dried at 180 °C. Values are means ± standard deviation of triplicate determinations. Means with different lowercase letters in the same column are significantly different at *p* < 0.05. HR—Hausner ratio; CI—Carr Index; D_4;3_—volume-weighted mean diameter; D_3;2_—volume/surface mean diameter; TSC—Total Solids Content.

**Table 2:** The effects of spray-drying conditions on solubility, water activity and changes in sulfhydryl group (–SH) and disulfide bond (–S–S–) contents of experimental powders.

Sample	Solubility (%)	a_w_ (—)	–SH (µmol/g)	–S–S– (µmol/g)
A—140 °C	58.01 ± 0.45 ^a^	0.45 ± 0.00 ^a^	67.39 ± 0.19 ^a^	19.68 ± 0.80 ^a^
B—160 °C	70.74 ± 0.81 ^b^	0.37 ± 0.01 ^b^	74.77 ± 1.08 ^b^	27.61 ± 0.35 ^b^
C—180 °C	63.96 ± 0.46 ^ab^	0.37 ± 0.00 ^c^	75.66 ± 0.55 ^b^	47.96 ± 2.05 ^c^

A—140 °C: sample spray-dried at 140 °C; B—160 °C: sample spray-dried at 160 °C; C—180 °C: sample spray-dried at 180 °C. Values are means ± standard deviation of triplicate determinations. Means with different lowercase letters in the same column are significantly different at *p* < 0.05. a_w_—water activity; –SH—sulfhydryl groups; –S–S–—disulfide bonds.

**Table 3:** The effect of spray-drying conditions on the early, advanced and final stages of Maillard reactions.

	Available Lysine (mg/g)	FIC (FI)	TRP (FI)	FAST (%)	Melanoidins (AU)	
					A_360_	A_420_
A—140 °C	1.26 ± 0.02 ^a^	60.73 ± 2.82 ^a^	49.91 ± 1.14 ^a^	121.73 ± 2.88 ^a^	0.621 ± 0.003 ^a^	0.248 ± 0.005 ^a^
B—160 °C	1.34 ± 0.04 ^a^	65.21 ± 2.54 ^b^	54.21 ± 2.28 ^b^	120.29 ± 0.60 ^a^	0.592 ± 0.009 ^b^	0.198 ± 0.005 ^b^
C—180 °C	1.76 ± 0.06 ^b^	61.59 ± 0.16 ^ab^	50.57 ± 1.11 ^a^	123.38 ± 1.23 ^a^	0.567 ± 0.013 ^c^	0.176 ± 0.002 ^c^

A—140 °C: sample spray-dried at 140 °C; B—160 °C: sample spray-dried at 160 °C; C—180 °C: sample spray-dried at 180 °C. Values are means ± standard deviation of triplicate determinations. Means with different lowercase letters in the same column are significantly different at *p* < 0.05. FIC—Fluorescent Intermediate Compounds; TRP—tryptophan; FAST—fluorescence of advanced Maillard products and soluble tryptophan.

**Table 4:** The physicochemical changes between the initial and the reconstituted samples.

Sample	pH (—)	TA (%)	RSC (mg/mL)	TPC (mg GAE/mL)	TFC (mg QE/mL)	TFAA (mg Gly/mL)
CPE	6.18 ± 0.01 ^a^	0.25 ± 0.01 ^a^	13.28 ± 0.02 ^a^	5.73 ± 0.26 ^a^	12.47 ± 0.01 ^a^	5.09 ± 0.04 ^a^
AR—140 °C	6.50 ± 0.01 ^b^	0.26 ± 0.01 ^a^	15.14 ± 0.11 ^b^	2.30 ± 0.09 ^b^	13.39 ± 0.06 ^b^	5.74 ± 0.02 ^b^
BR—160 °C	6.49 ± 0.00 ^b^	0.29 ± 0.01 ^a^	22.40 ± 0.00 ^c^	2.71 ± 0.15 ^c^	12.56 ± 0.09 ^c^	6.71 ± 0.04 ^c^
CR—180 °C	6.45 ± 0.01 ^c^	0.30 ± 0.01 ^a^	26.19 ± 0.07 ^d^	2.86 ± 0.20 ^d^	12.64 ± 0.05 ^d^	7.14 ± 0.01 ^d^

CPE: Camelina Press Cake Extract; AR—140 °C: reconstituted sample spray-dried at 140 °C; BR—160 °C: reconstituted sample spray-dried at 160 °C; CR—180 °C: reconstituted sample spray-dried at 180 °C. Values are means ± standard deviation of triplicate determinations. Means with different lowercase letters in the same column are significantly different at *p* < 0.05. TA—titrable acidity; RSC—Reducing Sugars Content; TPC—Total Polyphenols Content; TFC—Total Flavonoids Content; TFAA—Total Free Amino Acids.

**Table 5:** Antioxidant potential of Camelina Press Cake Extract before and after reconstitution.

Sample	DPPH (μmol Trolox/mL)	ABTS (μmol Trolox/mL)	FRAP (mg AAE/mL)	RP (μmol Trolox/mL)
CPE	4.03 ± 0.02 ^a^	10.61 ± 0.33 ^a^	7.18 ± 0.06 ^a^	4.71 ± 0.01 ^a^
AR—140 °C	4.27 ± 0.01 ^b^	12.59 ± 0.43 ^b^	6.61 ± 0.02 ^b^	3.37 ± 0.11 ^b^
BR—160 °C	4.18 ± 0.01 ^c^	9.38 ± 0.43 ^c^	6.12 ± 0.04 ^c^	2.46 ± 0.08 ^c^
CR—180 °C	4.44 ±0.03 ^d^	9.79 ± 0.18 ^d^	3.94 ± 0.05 ^d^	2.29 ± 0.01 ^d^

CPE: Camelina Press Cake Extract; AR—140 °C: reconstituted sample spray-dried at 140 °C; BR—160 °C: reconstituted sample spray-dried at 160 °C; CR—180 °C: reconstituted sample spray-dried at 180 °C. Values are means ± standard deviation of triplicate determinations. Means with different lowercase letters in the same column are significantly different at *p* < 0.05.

**Table 6:** Changes of the color coordinates between samples.

Powders						
Sample	L*	a*	b*	YI	WI	BI
A—140 °C	79.93 ± 0.00 ^a^	2.54 ± 0.00 ^a^	34.15 ± 0.02 ^a^	55.67 ± 0.04 ^a^	63.43 ± 0.02 ^a^	186.70 ± 0.00 ^a^
B—160 °C	78.87 ± 0.00 ^b^	1.81 ± 0.00 ^b^	33.56 ± 0.03 ^b^	60.79 ± 0.05 ^b^	66.08 ± 0.03 ^b^	186.43 ± 0.00 ^b^
C—180 °C	77.97 ± 0.00 ^c^	2.01 ± 0.00 ^c^	31.07 ± 0.03 ^c^	62.42 ± 0.05 ^c^	65.51 ± 0.03 ^c^	187.31 ± 0.00 ^c^
Reconstituted samples						
Sample	L*	a*	b*	YI	WI	ΔE
CPE	44.40 ± 0.01 ^a^	−5.56 ± 0.02 ^a^	17.29 ± 0.01 ^a^	55.63 ± 0.04 ^a^	80.37 ± 0.01 ^a^	Used as a standard
AR—140 °C	48.39 ± 0.01 ^b^	−5.07 ± 0.02 ^b^	19.55 ± 0.02 ^b^	57.72 ± 0.05 ^b^	78.56 ± 0.02 ^b^	4.61 ± 0.01 ^a^
BR—160 °C	48.84 ± 0.01 ^c^	−5.07 ± 0.01 ^b^	18.91 ± 0.01 ^c^	55.31 ± 0.02 ^c^	79.16 ± 0.01 ^c^	4.75 ± 0.00 ^b^
CR—180 °C	52.97 ± 0.00 ^d^	−5.25 ± 0.01 ^c^	21.79 ± 0.00 ^d^	58.77 ± 0.00 ^d^	76.56 ± 0.00 ^d^	9.68 ± 0.01 ^c^

A—140 °C: sample spray-dried at 140 °C; B—160 °C: sample spray-dried at 160 °C; C—180 °C: sample spray-dried at 180 °C; CPE: Camelina Press Cake Extract; AR—140 °C: reconstituted sample spray-dried at 140 °C; BR—160 °C: reconstituted sample spray-dried at 160 °C; CR—180 °C: reconstituted sample spray-dried at 180 °C; L*: lightness; a*: redness/greenness; b*: yellowness/blueness; YI: yellowness index; WI: whiteness index; BI: Browning Index; ΔE: total color difference. Values are means ± standard deviation of triplicate determinations. Means with different lowercase letters in the same column are significantly different at *p* < 0.05.

## Data Availability

Data are contained within the article.

## References

[1] Sapone A., Bai J.C., Ciacci C., Dolinsek J., Green P.H.R., Hadjivassiliou M., Kaukinen K., Rostami K., Sanders D.S., Schumann M. (2012). Spectrum of gluten-related disorders: Consensus on new nomenclature and classification. BMC Med..

[2] Lebwohl B., Sanders D.S., Green P.H.R. (2018). Coeliac Disease. Lancet.

[3] Molina-Infante J., Santolaria S., Sanders D.S., Fernández-Bañares F. (2015). Systematic review: Noncoeliac Gluten Sensitivity. Aliment. Pharmacol. Ther..

[4] Rowicka G., Czaja-Bulsa G., Chełchowska M., Riahi A., Strucińska M., Weker H., Ambroszkiewicz J. (2018). Oxidative and antioxidative status of children with celiac disease treated with a gluten free-diet. Oxid. Med. Cell Longev..

[5] Calabriso N., Scoditti E., Massaro M., Maffia M., Chieppa M., Laddomada B., Carluccio M.A. (2022). Non-celiac gluten sensitivity and protective role of dietary polyphenols. Nutrients.

[6] Kurasiak-Popowska D., Rynska B., Stuper-Szablewska K. (2019). Analysis of distribution of selected bioactive compounds in *Camelina sativa* from seeds to pomace and oil. Agronomy.

[7] Terpinc P., Polak T., Makuc D., Ulrih N.P., Abramovič H. (2012). The occurrence and characterisation of phenolic compounds in *Camelina sativa* seed, cake and oil. Food Chem..

[8] Sarv V., Trass O., Diosady L.L. (2017). Preparation and characterization of *Camelina sativa* protein isolates and mucilage. JAOCS.

[9] Li N., Qi G., Sun X.S., Wang D. (2016). Characterization of gum isolated from camelina seed. Ind. Crops Prod..

[10] Łopusiewicz Ł., Drozłowska E., Kwiatkowski P. (2022). Production and characterization of yogurt-like fermented beverage based on Camelina (*Camelina Sativa* L.) seed press cake. Appl. Sci..

[11] Usman I., Saif H., Imran A., Afzaal M., Saeed F., Azam I., Afzal A., Ateeq H., Islam F., Shah Y.A. (2023). Innovative applications and therapeutic potential of oilseeds and their by-products: An eco-friendly and sustainable approach. Food Sci. Nutr..

[12] Arrutia F., Binner E., Williams P., Waldron K.W. (2020). Oilseeds beyond oil: Press cakes and meals supplying global protein requirements. Trends Food Sci. Technol..

[13] Ancuţa P., Sonia A. (2020). Oil press-cakes and meals valorization through circular economy approaches: A review. Appl. Sci..

[14] Samborska K., Boostani S., Geranpour M., Hosseini H., Dima C., Khoshnoudi-Nia S., Rostamabadi H., Falsafi S.R., Shaddel R., Akbari-Alavijeh S. (2021). Green biopolymers from by-products as wall materials for spray drying microencapsulation of phytochemicals. Trends Food Sci. Technol..

[15] León-Martínez F.M., Rodríguez-Ramírez J., Medina-Torres L.L., Méndez Lagunas L.L., Bernad-Bernad M.J. (2011). Effects of drying conditions on the rheological properties of reconstituted mucilage solutions (*Opuntia ficusindica*). Carbohydr. Polym..

[16] Drozłowska E., Łopusiewicz Ł., Mężyńska M., Bartkowiak A. (2020). Valorization of flaxseed oil cake residual from cold-press oil production as a material for preparation of spray-dried functional powders for food applications as emulsion stabilizers. Biomolecules.

[17] Bustamante M., Laurie-Martínez L., Vergara D., Campos-Vega R., Rubilar M., Shene C. (2020). Effect of three polysaccharides (inulin, and mucilage from chia and flax seeds) on the survival of probiotic bacteria encapsulated by spray drying. Appl. Sci..

[18] Lipan L., Rusu B., Sendra E., Hernández F., Vázquez-Araújo L., Vodnar D.C., Carbonell-Barrachina Á.A. (2020). Spray drying and storage of probiotic-enriched almond milk: Probiotic survival and physicochemical properties. J. Sci. Food Agric..

[19] Jinapong N., Suphantharika M., Jamnong P. (2008). Production of instant soymilk powders by ultrafiltration, spray drying and fluidized bed agglomeration. J. Food Eng..

[20] Wilkowska A., Ambroziak W., Czyżowska A., Adamiec J. (2016). Effect of microencapsulation by spray-drying and freeze-drying technique on the antioxidant properties of blueberry (*Vaccinium myrtillus*) juice polyphenolic compounds. Pol. J. Food Nutr. Sci..

[21] Ibrahim F.M., Habbasha E. (2015). Chemical composition, medicinal impacts and cultivation of camelina (*Camelina sativa*): Review. Int. J. Pharm. Tech. Res..

[22] Łopusiewicz Ł., Drozłowska E., Tarnowiecka-Kuca A., Bartkowiak A., Mazurkiewicz-Zapałowicz K., Salachna P. (2020). Biotransformation of flaxseed oil cake into bioactive camembert-analogue using Lactic Acid Bacteria, *Penicillium camemberti* and *Geotrichum candidum*. Microorganisms.

[23] Horwitz W. (2000). Official Methods of Analysis of AOAC International.

[24] Drozłowska E., Bartkowiak A., Trocer P., Kostek M., Tarnowiecka-Kuca A., Łopusiewicz Ł. (2021). Formulation and evaluation of spray-dried reconstituted flaxseed oil-in-water emulsions based on flaxseed oil cake extract as emulsifying and stabilizing agent. Foods.

[25] Gong K.J., Shi A.M., Liu H.Z., Liu L., Hu H., Adhikari B., Wang Q. (2015). Emulsifying properties and structure changes of spray and freeze-dried peanut protein isolate. J. Food Eng..

[26] Gierczyński I., Guichard E., Laboure H. (2011). Aroma perception in dairy products: The roles of texture, aroma release, and consumer physiology. A review. Flavour Fragr. J..

[27] Carr L.R. (1965). Evaluating flow properties of solids. Chem. Eng..

[28] Hausner H.H. (1967). Friction conditions in a mass of metal powder. Int. J. Powder Metall..

[29] Łopusiewicz Ł., Waszkowiak K., Polanowska K., Mikołajczak B., Śmietana N., Hrebień-Filisińska A., Sadowska J., Mazurkiewicz-Zapałowicz K., Drozłowska E. (2022). The effect of yogurt and kefir starter cultures on bioactivity of fermented industrial by-product from *Cannabis sativa* production—Hemp press cake. Fermentation.

[30] Drozłowska E., Bartkowiak A., Trocer P., Kostek M., Tarnowiecka-Kuca A., Bienkiewicz G., Łopusiewicz Ł. (2021). The influence of flaxseed oil cake extract on oxidative stability of microencapsulated flaxseed oil in spray-dried powders. Antioxidants.

[31] Michalska A., Amigo-Benavent M., Zielinski H., Del Castillo M.D. (2008). Effect of bread making on formation of Maillard reaction products contributing to the overall antioxidant activity of rye bread. J. Cereal Sci..

[32] Zieliński H., Del Castillo M.D., Przygodzka M., Ciesarova Z., Kukurova K., Zielińska D. (2012). Changes in chemical composition and antioxidative properties of rye ginger cakes during their shelf-life. Food Chem..

[33] Łopusiewicz Ł., Drozłowska E., Siedlecka P., Mężyńska M., Bartkowiak A., Sienkiewicz M., Zielińska-Bliźniewska H., Kwiatkowski P. (2019). Development, characterization, and bioactivity of non-dairy kefir-like fermented beverage based on flaxseed oil cake. Foods.

[34] Barac M., Cabrilo S., Pesic M., Stanojevic S., Zilic S., Macej O., Ristic N. (2010). Profile and functional properties of seed proteins from six pea (*Pisum sativum*) genotypes. Int. J. Mol. Sci..

[35] Tong T., Liu Y.J., Kang J., Zhang C.M., Kang S.G. (2019). Antioxidant activity and main chemical components of a novel fermented tea. Molecules.

[36] Guo C., Yang J., Wei J., Li Y., Xu J., Jiang Y. (2003). Antioxidant activities of peel, pulp and seed fractions of common fruits as determined by FRAP assay. Nutr. Res..

[37] Reddy R.S., Ramachandra C.T., Hiregoudar S., Nidoni U., Ram J., Kammar M. (2014). Influence of processing conditions on functional and reconstitution properties of milk powder made from Osmanabadi goat milk by spray drying. Small Rumin. Res..

[38] Fitzpatrick J.J., Iqbal T., Delaney C., Twomey T., Keogh M.K. (2004). Effect of powder properties and storage conditions on the flowability of milk powders with different fat contents. J. Food Eng..

[39] Łopusiewicz Ł., Bogusławska-Wąs E., Drozłowska E., Trocer P., Dłubała A., Mazurkiewicz-Zapałowicz K., Bartkowiak A. (2021). The application of spray-dried and reconstituted flaxseed oil cake extract as encapsulating material and carrier for probiotic *Lacticaseibacillus rhamnosus* GG. Materials.

[40] Zhong C., Tan S., Zhou Z., Zhong X., Langrish T. (2023). Applications of aged powders of spray-dried whey protein isolate and ascorbic acid in the field of food safety. Dry. Technol..

[41] Akbarbaglu Z., Mahdi Jafari S., Sarabandi K., Mohammadi M., Khakbaz Heshmati M., Pezeshki A. (2019). Influence of spray drying encapsulation on the retention of antioxidant properties and microstructure of flaxseed protein hydrolysates. Colloids Surf. B Biointerfaces.

[42] Sadiq U., Gill H., Chandrapala J., Shahid F. (2023). Influence of spray drying on encapsulation efficiencies and structure of casein micelles loaded with anthraquinones extracted from *Aloe vera* plant. Appl. Sci..

[43] Boostani S., Aminlari M., Moosavi-nasab M., Niakosari M., Mesbahi G. (2017). Fabrication and characterisation of soy protein isolate-grafted dextran biopolymer: A novel ingredient in spray-dried soy beverage formulation. Int. J. Biol. Macromol..

[44] Fu G.M., Xu Z.W., Luo C., Xu L.Y., Chen Y.R., Guo S.L., Wu X.D., Wan Y. (2021). Modification of soy protein isolate by Maillard reaction and its application in microencapsulation of *Limosilactobacillus reuteri*. J. Biosci. Bioeng..

[45] Fang Y., Rogers S., Selomulya C., Chen X.D. (2012). Functionality of milk protein concentrate: Effect of spray drying temperature. Biochem. Eng. J..

[46] Liu D., Zhang J., Yang T., Liu X., Hemar Y., Regenstein J.M., Zhou P. (2019). Effects of skim milk pre-acidification and retentate pH-restoration on spray-drying performance, physico-chemical and functional properties of milk protein concentrates. Food Chem..

[47] Starowicz M., Zielinski H. (2017). Functional properties and Maillard reaction product formation in rye-buckwheat ginger cakes enhanced with rutin. Superfood and Functional Food—An Overview of Their Processing and Utilization.

[48] Langner E., Rzeski W. (2014). Biological properties of melanoidins: A review. Int. J. Food Prop..

[49] Souza C.R.F., Georgetti S.R., Salvador M.J., Vieira Fonseca M.J., Pereira Oliveira W. (2009). Antioxidant activity and physical-chemical properties of spray and spouted bed dried extracts of *Bauhinia forficate*. Braz. J. Pharm. Sci..

[50] Ali H., Al-khalifa A.R., Aboelsood W., Bareh G., Farouk A. (2019). Influence of spray-drying on improving the quality of dried carob juice. Qual. Assur. Saf..

[51] Wu G., Hui X., Mu J., Brennan M.A., Brennan C.S. (2021). Functionalization of whey protein isolate fortified with blackcurrant concentrate by spray-drying and freeze-drying strategies. Int. Food Res. J..

[52] Pérez-Gregorio M.R., Regueiro J., González-Barreiro C., Rial-Otero R., Simal-Gándara J. (2011). Changes in antioxidant flavonoids during freeze-drying of red onions and subsequent storage. Food Control.

[53] Rodrigues L.A., Matias A.A., Paiva A. (2021). Recovery of antioxidant protein hydrolysates from shellfish waste streams using subcritical water extraction. Food Bioprod. Process.

[54] Mrkìc V., Cocci E., Dalla Rosa M., Sacchetti G. (2006). Effect of drying conditions on bioactive compounds and antioxidant activity of broccoli (*Brassica oleracea* L.). J. Sci. Food Agric..

[55] Papoutsis K., Golding J.B., Vuong Q., Pristijono P., Stathopoulos C.E., Scarlett C.J., Bowyer M. (2018). Encapsulation of citrus by-product extracts by spray-drying and freeze-drying using combinations of maltodextrin with soybean protein and L-Carrageenan. Foods.

[56] Chen W., Chiu H.T., Feng Z., Maes E., Serventi L. (2021). Effect of spray-drying and freeze-drying on the composition, physical properties, and sensory quality of pea processing water (Liluva). Foods.

[57] Brishti F.H., Chay S.Y., Muhammad K., Ismail-Fitry M.R., Zarei M., Karthikeyan S., Saari N. (2020). Effects of drying techniques on the physicochemical, functional, thermal, structural, and rheological properties of mung bean (*Vigna radiata*) protein isolate powder. Int. Food Res. J..

[58] Tkacz K., Wojdyło A., Michalska-Ciechanowska A., Turkiewicz I.P., Lech K., Nowicka P. (2020). Influence carrier agents, drying methods, storage time on physico-chemical properties and bioactive potential of encapsulated sea buckthorn juice powders. Molecules.

[59] Koca N., Erbay Z., Kaymak-Ertekin F. (2015). Effects of spray-drying conditions on the chemical, physical, and sensory properties of cheese powder. J. Dairy Sci..

[60] Manjunatha S., Raju P.S. (2015). Rheological characteristics of reconstituted spray dried beetroot (*Beta vulgaris* L.) juice powder at different solid content, temperatures, and carrier materials. Int. Food Res. J..

[61] Rao M.A. (2014). Flow and functional models for rheological properties of fluid foods. Rheology of Fluid, Semisolid, and Solid Foods: Principles and Applications.

[62] Juszczak L., Witczak M., Fortuna T., Solarz B. (2010). Effect of temperature and soluble solids content on the viscosity of beetroot (*Beta vulgaris*) juice concentrate. Int. J. Food Prop..

